# Prognostic indicators of corneal ulcer clinical outcomes at a tertiary care center in the Bronx, New York

**DOI:** 10.1186/s12348-024-00392-3

**Published:** 2024-04-24

**Authors:** Sruthi Kodali, Behram Khan, Amanda M. Zong, Jee-Young Moon, Anurag Shrivastava, Johanna P. Daily, Richard P. Gibralter

**Affiliations:** 1 Department of Ophthalmology and Visual Sciences, Montefiore Medical Center, Albert Einstein College of Medicine, Bronx, NY USA; 2 Department of Epidemiology and Population Health (Biostatistics), Albert Einstein College of Medicine, Bronx, NY USA; 3 Department of Medicine (Infectious Diseases), Montefiore Medical Center, Albert Einstein College of Medicine, Bronx, NY USA

**Keywords:** Corneal Ulcer, Keratitis, Infectious Keratitis

## Abstract

**Purpose:**

Corneal ulcers frequently result in ocular morbidity and may lead to permanent visual impairment if severe or untreated. This study aims to evaluate the association of patient factors and ocular exam findings on clinical outcomes for patients diagnosed with a corneal ulcer at a tertiary care center in the Bronx, New York.

**Methods:**

A retrospective chart review was conducted on all ambulatory and admitted patients diagnosed with a corneal ulcer (identified using ICD-10 code H16.0) at Montefiore Medical Center, Bronx, NY between 2016–2022. Patient demographics, presence of known risk factors, characteristics of subsequent clinical course, and microbiological studies were noted. Clinical outcomes following treatment were longitudinally evaluated and categorized based upon the following criteria: 1) ‘No Surgical Intervention’: No severe complications or surgery required after presentation, 2) ‘Surgical Intervention’: Decline in BCVA with surgery required for a severe complication.

**Results:**

The search criteria identified 205 patients (205 eyes) with the diagnosis of a corneal ulcer. Mean age was 55.3 ± 21.1 years (mean ± SD). Mean ulcer area at presentation was 7 ± 10.5 mm^2^. Mean LogMAR at presentation was 1.2 ± 1, and following treatment, improved to 1.0 ± 1. ‘Surgical Intervention’ outcome was associated with advanced age (*p* = 0.005), presence of ocular surface disease (*p* = 0.008), central location of ulcer (*p* = 0.014), greater ulcer area at presentation (*p* = 0.003), worse visual acuity at presentation (*p* < 0.001), and isolation of fungi (*p* = 0.004).

**Conclusion:**

Identification of risk factors associated with a poor clinical prognosis can guide treatment and inform expectations for patients diagnosed with a corneal ulcer. Our study highlights the importance of timely diagnosis, work-up, and initiation of appropriate management, particularly in vulnerable populations where access to specialty care is logistically challenging.

## Background

Corneal ulcers are frequently associated with considerable ocular morbidity, and in severe cases, may lead to permanent visual impairment [1]. Ulcers develop from defects in the corneal epithelium, which allows for entry of infectious pathogens. Thus, certain risk factors such as corneal abrasions, contact lens use, and eye trauma can increase an individual’s susceptibility to corneal ulcer development [2]. Other known risk factors include presence of ocular surface disease, prior ocular surgery, diabetes mellitus, steroid use, systemic immunosuppression, manual labor, and lower education level. Though most corneal ulcers are infectious in nature, noninfectious etiologies include ocular injuries, chemical burns, and immune-mediated keratopathy [2]. Previous studies have shown older age, steroid use, poor visual acuity, and larger epithelial defects at initial presentation to be poor prognostic indicators [3–8].

Research on risk factors associated with corneal ulcer development and prognosis is limited in regions affected by low socioeconomic status (SES). Patients of low SES may independently be at greater risk for poor visual outcomes as prior studies have shown an association between low SES and poorer visual acuity [9–11].  Another analysis demonstrated the inverse to be true, where patients with higher income were almost one-third less likely to have functional blindness [12]. To our knowledge, this is the first study that evaluates prognostic factors of corneal ulcer outcome, microbiologic profile, and antibiotic treatment practices at Montefiore Medical Center (MMC), the largest healthcare provider for a highly diverse patient population in an area with low socioeconomic status (SES) in the Bronx, New York.

## Methods

### Study design

A retrospective chart review of our EMR database was conducted on all patients (ambulatory and admitted) diagnosed with a corneal ulcer (ICD-10: H16.0) between January 2016 and December 2022 at Montefiore Medical Center (MMC). This retrospective study was approved by the institutional review board (IRB) of Albert Einstein College of Medicine, Bronx, New York. This study adheres to the tenets of the declaration of Helsinki. Patients were excluded if a comprehensive chart review did not find clinical documentation of a corneal ulcer. Patients with incomplete data for some study variables were included in the relevant analyses based on the available information, but they were not omitted from the study overall.

### Data collection

Patient demographics, medical history, prior and current medication use, clinical characteristics of the corneal ulcer, and microbial culture results were extracted, along with history of diabetes (both type 1 and type 2), diabetic retinopathy, systemic immunosuppression, ocular surface disease (OSD), contact lens use, and history of eye surgery or trauma. Systemic immunosuppression was defined as: current use of immunosuppressive drugs (steroids, anti-rejection medications, and autoimmune treatments), malnutrition (BMI < 18.5), HIV/AIDS, or presence of autoimmune condition or malignancy. Patients met criteria for OSD if they had one or more of the following subtypes: dry eye syndrome, corneal epithelial defect, corneal abrasion, corneal erosion, limbal stem cell deficiency, keratopathy (neurotrophic, exposure, band), allergic conjunctivitis, keratoconjunctivitis sicca, cicatricial conjunctivitis, meibomian gland dysfunction/blepharitis, and prior chemical/thermal burns to the eye.

Furthermore, ulcer characteristics were recorded: laterality, size (calculated as length times width in mm^2^), location (central vs. peripheral), patient reported duration of ulcer-related symptoms, and antibiotic treatment regimen. In cases of bilateral ulcers, data was collected for the eye in which the first ulcer developed. All the aforementioned factors are the primary independent variables of the study. The primary dependent variables were baseline ulcer size, best corrected visual acuity (BCVA) at initial ophthalmic presentation, and clinical outcome. BCVA was measured using Snellen charts. Patients were divided into three distinct groups according to the initial ulcer size and into another three groups based on BCVA, with the divisions made using tertiles. Patients were divided into two groups for clinical outcome: surgical intervention and no surgical intervention.

The BCVA data was converted to LogMAR format and was analyzed by comparing initial measurements with those taken during the first visit occurring at least three months after treatment and up to one year following. 14 patients (6.8%) did not return after initial presentation and another 34 patients (16.6%) did not meet the three-month minimum follow-up requirement, resulting in their data being unavailable for inclusion in any subsequent analysis. For the remaining patients, clinical outcomes following treatment were longitudinally evaluated and categorized based upon the following criteria: 1) ‘No Surgical Intervention’: no surgery or severe complications after presentation, 2) ‘Surgical Intervention’: decline in BCVA with surgery required for a severe complication. Severe complications included endophthalmitis, hypopyon, vitritis, or corneal perforation along with presence of visually significant corneal scarring.

### Statistical analysis

Descriptive statistics (mean and SD, count and %) were computed to summarize the baseline characteristics of the patients. In addition, microbiological profile from corneal ulcer cultures was reported among all patients with positive cultures. We examined a bivariate association between risk factors and the clinical presentation of ulcer (size and visual acuity, separately) at baseline using a Mann–Whitney U-test for binary risk factors, a Kruskal–Wallis test for categorical variables with more than 2 categories, and a Spearman test for continuous variables. We also presented descriptive statistics (median and interquartile range (IQR) for continuous variables and count (%) for continuous variables. A bivariate association between risk factors and clinical outcome of ulcer (‘No Surgical Intervention’ vs. ‘Surgical Intervention’) was analyzed by a Fisher’s exact test for categorical variables and a Mann–Whitney test for continuous variables. Multiple testing correction was performed by Benjamini–Hochberg procedure and an FDR-corrected *P*-value < 0.1 was considered to be statistically significant. Statistical analysis was performed using the software R 4.2.0.

## Results

### Baseline characteristics

The mean age of the 205 patients identified with a corneal ulcer was 55.3 ± 21.1 years, of which 40.9% were male and 59% were female. With respect to self-identified race, 11.7% were White, 30.2% were Black, 2.4% were Asian, and 55.8% were Unknown. Included patients had a high proportion of either type I or type II diabetes mellitus (31.2%), and 4.9% had documented diabetic retinopathy. 41% of patients had pre-existing OSD, 25.4% had undergone previous eye surgery, 21% of patients were systemically immunosuppressed, and 11.7% had a history of trauma to the eye. With respect to contact lenses, 30.2% of patients reported prior use, of which 74% reported characteristics of poor hygiene (overnight wear, infrequent replacement, improper storage, and/or inadequate use of disinfecting solutions).

With respect to laterality, 52.2% of patients presented with an ulcer in the right eye and 47.8% in the left. Central corneal ulcers were seen in 45.9% of patients, with the remainder having documentation of a peripheral location. Interestingly, 20.4% of patients had documentation of steroid eye drop use at some point prior to ulcer development. LogMAR at presentation was 1.2 ± 1 and area was 7 ± 10.5 mm. The average patient-reported start of symptoms was 5.9 ± 8.1 days prior to ophthalmic presentation.

### Microbiologic profile and treatment practices

Of 205 total corneal ulcers, 65.4% (134) were cultured, of which 67.9% (91) showed growth of at least one microbiological organism. 28/91 (30.8%) showed growth of >1 organism. Among all patients with positive culture (*n* = 91), the most common species were Staphylococcus (*n* = 62), followed by Streptococcus (*n* = 20), and Pseudomonas (*n* = 18) species. Among contact lens users (*n* = 63), 58.7% (37) were cultured, of which 64.9% (24) showed growth of >1 organism. The most common species were Staphylococcus (n = 16), followed by Pseudomonas (*n* = 10), and Streptococcus (*n* = 2) (Table 1). 96.1% of patients received treatment with one or more topical antibiotics including Vancomycin (51%), Tobramycin (50%), Ofloxacin (20.9%), Moxifloxacin (19.9%), Erythromycin (14.6%), and Neomycin (6.3%).

**Table 1:** Microbiological profile of corneal ulcer cultures

Variable	Number among all patients (*n* = 205)	Number among contact lens users (*n* = 63)
**Cultured**	134 (65.4%)	37 (58.7%)
**Organism growth**^**a**^	91 (44.4%)	24 (38.1%)
**Pseudomonas aeruginosa**	18	10
**Staphylococcus**	62	16
** Coagulase-negative**	5	2
** Epidermis**	31	8
** Staphylococcus aureus**		
** Methicillin-sensitive**	12	3
** Methicillin-resistant**	1	0
** Lugdunensis**	4	1
** Warneri**	2	0
** Hominis**	2	2
** Haemolyticus**	2	0
** Capissi**	2	0
** Simulans**	1	0
**Streptococcus**	20	2
** Viridans**	6	2
** Pneumoniae**	7	0
** Mitis/oralis**	3	0
** Pyogenes**	3	0
** Dysgalactiae**	1	0
**Candida**	2	0
** Albicans**	1	0
** Glabrata**	1	0
**Moraxella**	6	0
** Nonliquefaciens**	3	0
** Lacunata**	3	0
**Bacillus**	3	0
** Subtilis**	2	0
** Licheniformis**	1	0
**Haemophilus**	3	1
** Parahaemolyticus**	2	0
** Influenza**	1	1
**Corynebacterium**	5	1
** Macginleyi**	4	1
** Pseudodiphtheriticum**	1	0
**Aspergillus**	2	1
** Versicolor**	1	0
** Flavus**	1	1
**Other**	15	2
** Strenotrophomas**	1	1
** maltophilia**	1	0
** Staph-like organism**	1	0
** Serratia marcescens**	1	0
** Rothia mucilaginosa**	1	0
** Rhizobium species**	1	0
** Kocuria species**	1	0
** Proteus mirabilis**	1	0
** Granulicatella adiacens**	1	0
** Enterobacter cloacae**	1	1
** Enterococcus faecalis**	1	0
** Pasteurella bettyae**	1	0
** Dolosigranulum pigrum**	1	0
** Acinetobacter species**	1	0
** Ursingii species**	1	0

^a^Several cultures grew > 1 organism

### Factors associated with ulcer size and visual acuity at baseline

Significant variables associated with greater ulcer size at baseline were older age (*p* < 0.001), central ulcer (*p* < 0.001), higher LogMAR at presentation (*p* < 0.001), and isolation of gram-negative rods (*p* < 0.001) (Table 2). Following multiple correction testing, these associations remain statistically significant.

**Table 2:** Association between risk factors for corneal ulcer development and ulcer size at presentation (*N* = 156)

Variable	Size < 1 (*n* = 32)	1 < = size < 5 (*n* = 69)	Size > = 5 (*n* = 55)	*P*-value	Corrected *P*-value	Spearman Correlation
***Gender***						
Male	10 (31.2%)	21 (29.4%)	26 (47.3%)	0.190	0.342	
Female	22 (68.8%)	48 (70.6%)	29 (52.7%)			
***Race***						
White	1 (3.1%)	7 (10.1%)	6 (10.9%)	0.066	0.239	
Black	9 (28.1%)	19 (27.5%)	21 (38.2%)			
Other	22 (68.8%)	43 (62.3%)	28 (50.9%)			
***Age***						
Median	47	52.5	62	** < 0.001**	** < 0.001**	0.35 (0.2–0.48)
IQR	31.8–57.8	35.8–63.5	53–75.5			
***Diabetes***	9 (28.1%)	18 (26.5%)	20 (36.4%)	0.274	0.411	
***Systemic Immunosuppression***	5 (15.6%)	12 (17.6%)	16 (29.1%)	0.088	0.245	
***Ocular Surface Disease***	13 (40.6%)	24 (34.8%)	29 (52.7%)	0.118	0.245	
***Previous Eye Surgery***	5 (15.6%)	16 (23.5%)	14 (25.5%)	0.358	0.473	
***History of Trauma***	6 (19.4%)	9 (13%)	6 (11.1%)	0.417	0.501	
***Inappropriate Contact Lens Use***	11 (84.6%)	16 (64%)	13 (76.5%)	0.516	0.516	
***Steroid Eye Drop Use (Local Immunosuppression)***	4 (12.5%)	14 (20.6)	14 (25.5%)	0.122	0.245	
***Corneal Ulcer Location***						
Central	15 (41.4%)	25 (35.3%)	39 (69.2%)	** < 0.001**	** < 0.001**	
Peripheral	17 (58.6%)	44 (64.7%)	16 (30.8%)			
***Patient Reported Start of Symptoms***^***a***^						
Median	2.5	3	3	0.249	0.408	0.1 (-0.07–0.26)
IQR	1–4	1.8–6.2	1–7			
***Log MAR (at presentation)***						
Median	0.3	0.5	1.9	** < 0.001**	** < 0.001**	0.55 (0.43–0.65)
IQR	0.1–0.5	0.3–1.9	1.1–2.3			
***Among Patients with Cultured Result (n* = *108)***	***n* = 17**	***n* = 39**	***n* = 52**			
***Positive Culture***	12 (70.6%)	25 (64.1%)	38 (73.1%)	0.469	0.516	
***Gram- Positive Cocci Presence***	11 (64.7%)	19 (48.7%)	25 (48.1%)	0.368	0.473	
***Gram-Negative Rod Presence***	0 (0%)	6 (15.4%)	20 (38.5%)	** < 0.001**	** < 0.001**	
***Gram-Positive Rod Presence***	1 (5.9%)	1 (2.6%)	3 (5.8%)	0.492	0.516	
***Fungi Presence***	0 (0%)	1 (2.6%)	3 (5.8%)	0.112	0.245	

Values are count (%) for categorical variables and median (IQR) for continuous variables

*P*-value by Mann–Whitney U-test for binary variables and by a Kruskal–Wallis test for categorical variables with more than 2 categories, and a Spearman test for continuous variables. The corrected *P*-value represents the *p*-value after multiple testing correction was performed

^a^Number of Days Prior to Presentation

Significant variables associated with worse visual acuity (higher LogMAR) at baseline were advanced age (*p* < 0.001), diabetes (*p* < 0.001), OSD (*p* = 0.004), previous eye surgery (*p* < 0.001), use of steroid eye drops prior to ulcer development (*p* = 0.002), central ulcer location (*p* < 0.001), earlier patient-reported start of symptoms prior to initial ophthalmic evaluation (*p* = 0.006), greater ulcer area (*p* < 0.001), and isolation of gram- negative rods (*p* = 0.023) (Table 3). Following multiple correction testing, these associations remain statistically significant.

**Table 3:** Association between Risk Factors for Corneal Ulcer Development and Visual Acuity at Presentation (*N* = 193)

Variable	LogMAR < 0.4 (*n* = 59)	0.4 < = logMAR < 1.9 (*n* = 56)	logMAR > = 1.9 (*n* = 78)	*P*-value	Corrected *P*-value	Spearman Correlation
***Gender***						
Male	25 (41.4%)	22 (32.1%)	36 (46.2%)	0.255	0.354	
Female	34 (58.6%)	38 (67.9%)	42 (53.8%)			
***Race***						
White	11 (18.6%)	4 (7.1%)	5 (6.4%)	0.155	0.233	
Black	15 (25.4%)	16 (28.6%)	29 (37.2%)			
Other	33 (56%)	36 (64.3%)	44 (56.4%)			
***Age***						
Median	46.5	51	65	** < 0.001**	** < 0.001**	0.4 (0.28–0.51)
IQR	32.2–59.2	35.2–67.2	55–78.8			
***Diabetes***	13 (22.4%)	12 (21.4%)	38 (48.7%)	** < 0.001**	**0.002**	
***Systemic Immunosuppression***	8 (13.8%)	14 (25%)	19 (24.4%)	0.288	0.370	
***Ocular Surface Disease***	16 (27.1%)	23 (41.1%)	43 (55.1%)	**0.004**	**0.010**	
***Previous Eye Surgery***	9 (15.5%)	10 (17.9%)	33 (42.3%)	** < 0.001**	** < 0.001**	
***History of Trauma***	10 (17.2%)	6 (10.9%)	8 (10.3%)	0.492	0.554	
***Inappropriate Contact Lens Use***	16 (69.6%)	14 (70%)	12 (80%)	0.594	0.629	
***Steroid Eye Drop Use (Local Immunosuppression)***	4 (6.8%)	13 (23.2%)	24 (30.8)	**0.002**	**0.005**	
**Corneal Ulcer Location**						
Central	18 (22.6%)	29 (44.9%)	54 (69.2%)	** < 0.001**	** < 0.001**	
Peripheral	41 (77.4%)	27 (55.1%)	24 (30.8%)			
***Patient Reported Start of Symptoms***^***a***^						
Median	2	3	4	**0.006**	**0.014**	0.22 (0.06–0.36)
IQR	1–5	2–4	2–14			
**A*rea***						
Median	0.6–2.1	0.9–4	3.1–20	** < 0.001**	** < 0.001**	0.55 (0.43–0.65)
IQR	**1**	1.1	9.8			
***Among patients with culture (n* = *126)***	***n* = 23**	***n* = 38**	***n* = 65**			
***Positive Culture***	14 (60.9%)	25 (65.8%)	48 (73.8%)	0.066	0.119	
***Gram- Positive Cocci Presence***	9 (39.1%)	23 (60.5%)	32 (49.2%)	0.649	0.649	
***Gram-Negative Rod Presence***	5 (21.7%)	4 (10.5%)	21 (32.3%)	**0.023**	**0.046**	
***Gram-Positive Rod Presence***	1 (4.3%)	1 (2.6%)	4 (6.2%)	0.365	0.437	
***Fungi Presence***	0 (0%)	0 (0%)	5 (7.7%)	0.124	0.203	

Values are count (%) for categorical variables and median (IQR) for continuous variables

*P*-value by Mann–Whitney U-test for binary variables and by a Kruskal–Wallis test for categorical variables with more than 2 categories, and a Spearman test for continuous variables. The corrected *P*-value represents the *p*-value after multiple testing correction was performed

^a^Number of Days Prior to Presentation

### Risk factors for clinical prognosis of ulcer

At the first appointment following three months of treatment initiation (mean of 132.9 days with a standard deviation of 79.5 days), the average LogMAR vision was 1.0 ± 1, indicating a 1-line improvement in Snellen visual acuity from 20/317 to 20/200. 127 patients (80.9%) were categorized in the 'No Surgical Intervention' outcome group, while 30 patients (19.1%) were in the 'Surgical Intervention' group. With respect to complications, 74 (36.1%) patients had some degree of corneal scarring, 25 (12.2%) had hypopyon, 10 (4.9%) had vitritis, 3 (1.5%) had corneal perforation, and 3 (1.5%) had endophthalmitis.

**Fig. 1:**
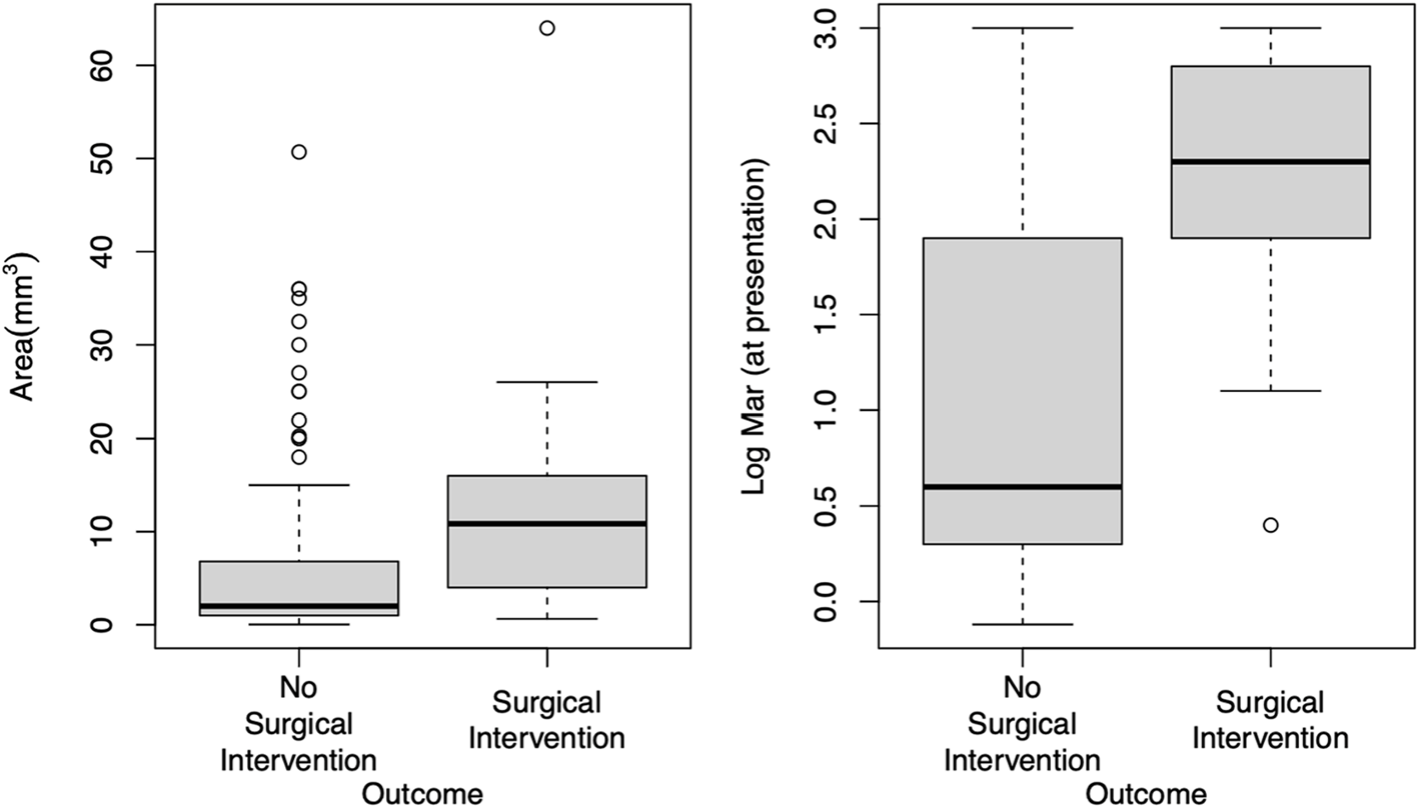
Area and LogMAR (initial presentation) by outcome

Significant variables associated with ‘Surgical Intervention’ outcome group (*n* = 30) were advanced age (*p* = 0.005), OSD (*p* = 0.008), central location (*p* = 0.014), greater ulcer area (*p* = 0.003), higher LogMAR at presentation (*p* < 0.001), and presence of fungi (*p* = 0.004) (Table 4). These associations remain statistically significant following multiple correction testing. Figure 1 displays the effects of area and LogMAR at initial presentation on outcome.

**Table 4:** Risk factors for corneal ulcer development associated with clinical outcomes (No Surgical Intervention vs. Surgical Intervention)

Variable	No Surgical Intervention (*N* = 127)	Surgical Intervention (*N* = 30)	*P*-value	Corrected *P*-value
***Gender***				
Male	55 (43.7%)	10 (33.3%)	0.410	0.649
Female	71 (56.3%)	20 (66.7%)		
***Race***				
White	14 (11%)	2 (6.7%)	0.541	0.734
Black	37 (29.1%)	12 (40%)		
Other	76 (59.8%)	16 (53.3%)		
***Age***				
Median	57	62.5	**0.005**	**0.024**
IQR	40.5–73.8	58.2–84.8		
***Diabetes***	45 (35.7%)	15 (50%)	0.210	0.443
***Systemic Immunosuppression***	31 (24.6%)	6 (20%)	0.811	1.000
***Ocular Surface Disease***	54 (42.5%)	21 (70%)	**0.008**	**0.031**
***Previous Eye Surgery***	37 (29.4%)	14 (46.7%)	0.084	0.229
***History of Trauma***	15 (12%)	2 (6.7%)	0.529	0.734
***Inappropriate Contact Lens Use***	25 (67.6%)	3 (75%)	1.000	1.000
***Use of Steroid Eye Drops***	30 (23.6%)	11 (36.7%)	0.167	0.397
***Type of Corneal Ulcer***				
Central	50 (43.5%)	18 (72%)	**0.014**	**0.045**
Peripheral	65 (56.5%)	7 (28%)		
***Pt reported start of symptoms***^***a***^				
Median	3	4	0.948	1.000
IQR	2–7	1.8–7		
***Ulcer area***				
Median	2	10.8	**0.003**	**0.024**
IQR	1–6.8	4–16		
***Log MAR (at presentation)***				
Median	0.6	2.3	** < 0.001**	** < 0.001**
IQR	0.3–1.9	1.9–2.8		
***Among patients with culture (n* = *108)***	***n* = 80**	***n* = 28**		
***Positive Culture***	57 (71.2%)	20 (71.4%)	1.000	1.000
***Gram-Positive Cocci Presence***	44 (55%)	12 (42.9%)	0.282	0.536
***Gram-Negative Rod Presence***	18 (22.5%)	9 (32.1%)	0.321	0.554
***Gram-Positive Rod Presence***	3 (3.8%)	1 (3.6%)	1.000	1.000
***Fungi Presence***	0 (0%)	4 (14.3%)	**0.004**	**0.024**

Values are count (%) for categorical variables and median (IQR) for continuous variables

*P*-value by a Fisher’s exact test for categorical variables and a Mann–Whitney U-test for continuous variables. The corrected *P*-value represents the *p*-value after multiple testing correction was performed

^a^Number of Days Prior to Presentation

## Discussion

In our analysis of patients diagnosed with a corneal ulcer, we determined prognostic factors for clinical outcomes that necessitated surgical intervention, Additionally, we documented population-specific demographics, associated risk factors, microbiologic profile, and treatment practices in the Bronx. A considerable proportion of patients had known risk factors for corneal ulcer development [7, 8, 13, 14], including pre-existing ocular surface disease (41%), type 1 or type 2 diabetes (31.2%), contact lens use (30.7%), previous eye surgery (25.4%), systemic immunosuppression (21%), steroid eye drop use (20.5%), and history of eye trauma (11.7%). Jeng et al. [15] reported that in a population with ulcerative keratitis in Northern California, the top three risk factors were: contact lens use (55%), OSD (19.2%), and trauma (11.9%). Another study by Truong et al. [16] studied a population of patients in Dallas, Texas with microbial keratitis, reporting contact lens use (41%), OSD (28%), trauma (17%), and topical steroid use (4%) as the top four risk factors. Compared to these studies, our population in the Bronx shows a fewer percentage of contact lens users (30.2%), but a much higher proportion of patients with OSD (41%).

Our results further elucidate contributing factors to the clinical severity of ulcers characterized by larger size and worse visual acuity at presentation. These factors were largely intuitive, and included increased age, central location, and isolation of gram-negative rods, consistent with previous studies [17–19]. Factors associated with worse visual acuity at presentation, but not necessarily with increased ulcer size, included presence of OSD, use of steroid eye drops, history of previous eye surgery, and longer duration of symptoms prior to evaluation.

Interestingly, patients with longer duration of symptoms prior to evaluation had worse visual acuity at presentation, indicating that a delay in diagnosis may lead to worse clinical presentation. Poor health care literacy compounded with challenges logistically accessing acute subspecialty care undoubtedly play an important role in these delays. Indeed, studies have demonstrated that many patients show poor knowledge of complications associated with corneal ulcers and when to seek medical attention [20], a finding which is even more pronounced in contact lens users [21]. Another explanation is that patients may first present to an intermediary location (i.e. emergency department, general practitioner) potentially delaying ophthalmologic evaluation. An audit performed on corneal abrasion management at an acute care setting in the United Kingdom revealed that without proper training for management of ophthalmic conditions, many practitioners did not feel confident in managing corneal abrasions, with only 41.2% of cases appropriately discharged [22]. Furthermore, delayed presentation can result in poorer clinical outcomes and can be more costly if surgery is required due to complications [23]. In our study, delayed presentation was significantly associated with initial clinical severity, but not significantly associated with worse clinical prognosis. However, patients that had outcomes requiring surgical intervention had a longer duration of symptoms prior to presentation, on average, compared to patients with outcomes that did not require surgical intervention, though this association was not significant. An interplay of the aforementioned factors may be involved in the delayed presentation to Ophthalmology leading to increased patient morbidity on presentation. This may be especially true in the Bronx, where patients are more likely to present to intermediaries due to high utilization of the emergency department (42%) [24]. Prompt access to subspecialty care equipped to diagnose and treat complex corneal pathologies can lead to more efficiently and effectively delivered care, while increasing patient comfort.

Overall, we saw a small improvement in visual acuity by an average of 0.2 LogMAR, from 1.2 ± 1 (20/317 Snellen) initially to 1.0 ± 1 (20/200 Snellen) following treatment. A multitude of prognostic indicators for visual outcome were elucidated in our study, including advanced age, worse visual acuity and greater ulcer size at presentation, presence of OSD, central location of ulcer, and presence of fungi. These findings are concordant with Khoo et al. [3], who also demonstrated that older age, poorer visual acuity, and larger epithelial defects at initial presentation were associated with poor patient outcomes. Furthermore, Green et al. [25] showed that patients with OSD were 4.1 times more likely to have poor outcomes.

In our study, although patients with positive cultures for gram negative rods such as Pseudomonas initially presented with greater clinical severity, these organisms were not associated with worse clinical outcomes post-treatment, indicating perhaps that these patients were more likely to respond to antibiotics compared to other types of bacterial ulcers [26]. In contrast, patients positive for fungal infection had worse clinical outcomes, consistent with the known therapeutic challenges and associations with greater complications such as perforation [27–30].

In our study, 65.8% of all corneal ulcers were cultured, of which 67.4% showed isolation of at least one microbiological organism. This rate of culture positivity is consistent with prior reports in the literature [27, 31, 32]. However, given the high utilization of emergency departments in our population, pretreatment with antibiotics prior to ophthalmology consultation may have skewed the culture results, yielding an artificially high percentage of ulcers that were culture negative. Given this limitation, a sub-analysis between infectious vs. non-ulcers was not conducted. Staphylococcus species was the most isolated organism overall, concordant with other studies performed in the United States [33, 34]. Interestingly, Staphylococcus species was also the most common organism in contact lens users, followed closely by Pseudomonas. It is well documented in the literature that Pseudomonas is the most common cause of corneal ulcers in contact lens users [35–39], although Staphylococci is also very common [40]. These discordant rates seen may be secondary to the relatively small sample size of contact lens users with positive cultures in our study.

### Limitations

One limitation of our study is the retrospective study design. Data collected is limited to the accuracy of the information recorded in the chart, and not all variables of interest were available for every patient. Another drawback is that BCVA was not uniformly measured and documented at ophthalmologic encounters. Data on baseline visual acuity prior to ulcer development and BCVA at initial medical presentation (i.e., emergency department) was not universally available.

## Conclusion

Identification of risk factors that impart a poor clinical prognosis can help guide treatment and inform patient expectations. In our study, risk factors associated with worse clinical outcomes were presence of fungi, worse visual acuity and greater ulcer size at presentation, central location of ulcer, presence of ocular surface disease, and advanced age. Pretreatment with antibiotics from referring providers may have impacted culture results and therapeutic decisions at the time of initial ophthalmologic evaluation. Thus, timely diagnosis and prompt referral to subspecialty care is crucial to optimize clinical outcomes, particularly in underserved communities where emergency department utilization is high. Given the high potential for vision loss and associated comorbidity, urgent evaluation, diagnosis, and treatment remain cornerstones in the management of corneal ulcers.

## Data Availability

The datasets used and/or analyzed during the current study are available from the corresponding author on reasonable request.

## Abbreviations

OSD: Ocular Surface Disease
BCVA: Best Corrected Visual Acuity
